# Conservative versus surgical treatment of foot drop in peroneal nerve entrapment: rationale and design of a prospective, multi-centre, randomized parallel-group controlled trial

**DOI:** 10.1186/s13063-022-07009-x

**Published:** 2022-12-30

**Authors:** Christophe Oosterbos, Sofie Rummens, Kris Bogaerts, Sophie Hoornaert, Frank Weyns, Annie Dubuisson, Robin Lemmens, Tom Theys

**Affiliations:** 1 Research Group experimental Neurosurgery and Neuroanatomy and the Leuven Brain Institute, KU Leuven, Leuven, Belgium; 2 Department of Neurosurgery, University Hospitals Leuven, Leuven, Belgium; 3 Department of Physical Medicine and Rehabilitation, University Hospitals Leuven, Leuven, Belgium; 4 Locomotor and Neurological disorders, KU Leuven, Leuven, Belgium; 5 Department of public health and critical care, I-BioStat, KU Leuven, Belgium and I-BioStat, UHasselt, Hasselt, Belgium; 6 Department of Neurosurgery, Ziekenhuis Oost-Limburg, Genk, Belgium; 7 Neurosciences, Faculty of Medicine and Life Sciences, UHasselt, Hasselt, Belgium; 8 Department of Neurosurgery, University Hospitals Liège, Liège, Belgium; 9 Department of Neurosciences, Experimental Neurology, KU Leuven – University of Leuven, Leuven, Belgium; 10 VIB, Center for Brain & Disease Research, Laboratory of Neurobiology, Leuven, Belgium; 11 Department of Neurology, University Hospitals Leuven, Leuven, Belgium

**Keywords:** Randomized controlled trial, Foot drop, Peroneal nerve, Neurolysis, Conservative treatment, Protocol design

## Abstract

**Background:**

High-quality evidence is lacking to support one treatment strategy over another in patients with foot drop due to peroneal nerve entrapment. This leads to strong variation in daily practice.

**Methods/design:**

The FOOTDROP (Follow-up and Outcome of Operative Treatment with Decompressive Release Of The Peroneal nerve) trial is a randomized, multi-centre study in which patients with peroneal nerve entrapment and persistent foot drop, despite initial conservative treatment, will be randomized 10 (± 4) weeks after onset between non-invasive treatment and surgical decompression. The primary endpoint is the difference in distance covered during the 6-min walk test between randomization and 9 months later. Time to recovery is the key secondary endpoint. Other secondary outcome measures encompass ankle dorsiflexion strength (MRC score and isometric dynamometry), gait assessment (10-m walk test, functional ambulation categories, Stanmore questionnaire), patient-reported outcome measures (EQ5D-5L), surgical complications, neurological deficits (sensory changes, motor scores for ankle eversion and hallux extension), health economic assessment (WPAI) and electrodiagnostic assessment.

**Discussion:**

The results of this randomized trial may elucidate the role of surgical decompression of the peroneal nerve and aid in clinical decision-making.

**Trial registration:**

ClinicalTrials.gov NCT04695834. Registered on 4 January 2021.

**Supplementary Information:**

The online version contains supplementary material available at 10.1186/s13063-022-07009-x.

## Background

Peroneal neuropathy is the most common mononeuropathy in the lower limb [1, 2] frequently causing foot drop, leading to gait difficulties and an increased risk of falling [3]. As the aetiology of peroneal neuropathy is broad and treatment strategies potentially differ accordingly, we previously proposed to classify peroneal neuropathies as idiopathic, idiopathic with established risk factors (e.g. leg crossing, squatting, weight loss, kneeling, metabolic disorders, bracing, positioning during surgery…) and non-idiopathic peroneal neuropathies (e.g. trauma, iatrogenic, cysts and tumours…) [4]. The term peroneal nerve entrapment will be used to refer to idiopathic peroneal neuropathies with and without established risk factors and is the subject of the randomized trial, discussed in this paper.

A scoping review was conducted to systematically map and discuss existing literature on (non-)invasive treatment of peroneal nerve entrapment and to identify knowledge gaps to guide further research [4]. The findings indicated a paucity of high-level evidence, since most studies were (retrospective) case series. No guidelines are available and studies comparing non-invasive treatment and neurolysis are lacking. Good outcome has been reported for both treatment strategies, with percentages ranging from 0 to 100% for conservatively treated patients [5–17] and 40% to 100% after surgery [2, 9, 10, 12, 13, 17–29]. Good outcome was not uniformly defined making direct comparison of studies even more difficult. In most reports, good outcome was defined in terms of recovery for ankle dorsiflexion muscle strength [4].

The absence of evidence to support any treatment strategy translates to important variations in daily practice. To map these differences in patient management, an international survey among specialists was conducted [30]. The survey was distributed throughout various national and international scientific societies. Responses from 181 medical specialists worldwide were analysed. Based on this survey, we found important differences in attitudes not only between, but also within specialisms. Treatment strategies range from early neurolysis to prolonged non-invasive treatment without a place for surgical treatment strategies. Furthermore, no health-economic data on the cost-effectiveness of non-invasive versus invasive treatment strategies were reported.

A randomized controlled trial is warranted to collect high-quality data on patient management and to determine a possible role for surgical decompression in improving outcomes. This article discusses the design of that randomized controlled trial. Publication of this protocol will aid in research transparency and protocol adherence during and after the trial [31]. The full title of the trial is ‘A prospective, multi-centre, randomized, parallel-group controlled trial to compare conservative versus surgical treatment of foot drop in peroneal nerve entrapment.’ The trial acronym is FOOTDROP (*F*ollow-up and *O*utcome of *O*perative *T*reatment with *D*ecompressive *R*elease *O*f the *P*eroneal nerve). The study protocol was piloted in a limited number of centres [6] to assess feasibility. Pilot study results were taken into account to shape the trial design as discussed in this manuscript.

## Methods/design

The FOOTDROP trial is a prospective, multi-centre, randomized, parallel-group controlled trial. The main objective of the trial is to establish whether foot drop, caused by peroneal nerve entrapment, recovers better within 9 months after decompressive surgery compared to prolonged conservative treatment. Important secondary objectives aim to compare the quality of life data between surgically and conservatively treated patients, to collect follow-up electrodiagnostic data in both patient groups, to assess the evolution of gait impairment and to evaluate the cost-effectiveness of both treatment strategies. The study protocol closely adheres to the SPIRIT 2013 statement guidelines [32]. The SPIRIT checklist is completed for the FOOTDROP study and is available in Appendix 1. The World Health Organization Trial Registration Data Set is available in Appendix 2.

Study results will help establish the place of neurolysis in the treatment of peroneal nerve entrapment and aid physicians to counsel patients on treatment and prognosis.

**Fig. 1:**
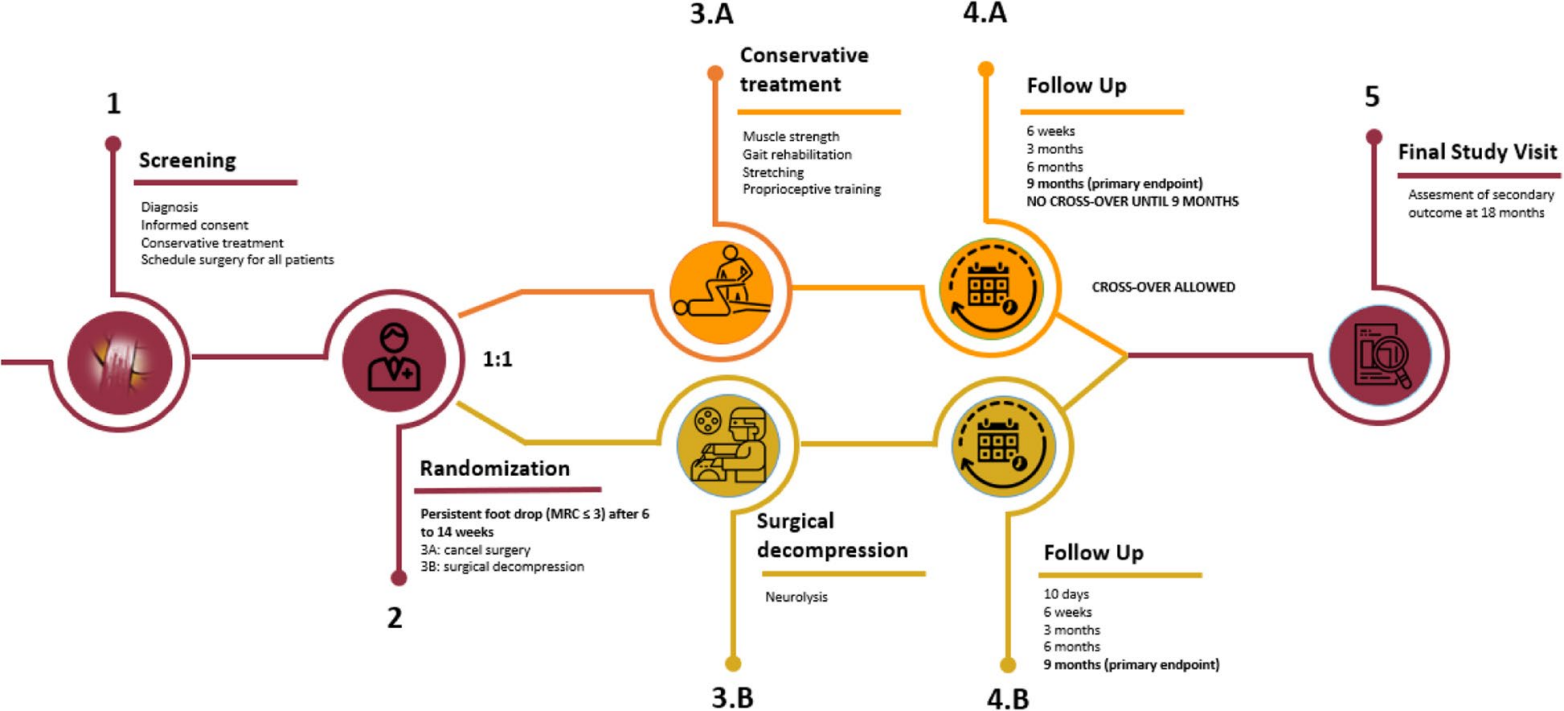
Trial flow

The main research question will be answered 9 months after randomization and the complete follow-up will last 18 months (after randomization). The study protocol was approved by all local Medical Ethics Committees. Figure 1 visualizes the trial flow.

### Trial registration

The trial is registered on ClinicalTrials.gov, identifier NCT04695834. KCE trials number is KCE19-1232 and the local sponsor study number is S62895.

### Patients

All adult patients with foot drop (defined as Medical Research Council Score (MRC) ≤ 3 for ankle dorsiflexion) and electrodiagnostic (EDX) confirmation of peroneal nerve entrapment at the level of the fibular head can be included if the presence of a compressive mass at the level of the fibular head is excluded by imaging (ultrasound, MRI) and if the eligibility criteria (see Table 1) are met. The multi-centre design with the involvement of over 20 hospitals throughout Belgium and several Dutch centres will result in a representative study population. A list of participating centres is included in the study protocol.

**Table 1:** Eligibility criteria

**Inclusion criteria**	
- EDX-documented peroneal nerve entrapment with persisting (10 ± 4 weeks) foot drop (MRC ≤ 3)	
- Age ≥ 18 years	
- Imaging (ultrasound/MRI) to exclude a compressive mass at the level of the fibular head	
- Written informed consent	
**Exclusion criteria**	
- Posttraumatic/iatrogenic peroneal nerve injury	
- Peroneal neuropathy due to compressive mass	
- Peroneal neuropathy at other sites than fibular head	
- Bilateral peroneal nerve entrapment	
- Psychiatric illness	
- Pregnancy	
- Previous foot drop	
- Permanently bedridden subjects	
- Neurological/musculoskeletal history with impact on assessment and/or gait analysis	
- Incapacitated to participate in physiotherapy programme (mental/physical illness)	
- Planned (e)migration within 1 year after randomization	

After diagnosis, patients will undergo conservative, non-invasive treatment. Inclusion will occur at the screening visit, organized by the local study team as soon as possible. During the screening visit, the patient’s history will be taken and a standardized neurological examination is performed. After a successful screening, the treating physician will obtain informed consent.

To identify all possible trial candidates, a local multidisciplinary study team (including physical medicine, neurology, neurosurgery, orthopaedic surgery and plastic and reconstructive surgery) is established. Active involvement of the different physicians is key to trial success.

### Treatment allocation

All patients with a foot drop due to peroneal nerve entrapment can be randomized 6 to 14 weeks after onset if the inclusion criteria are met (see Table 1). Patients are 1:1 randomized between prolonged non-invasive treatment and neurolysis of the peroneal nerve within 1 week after randomization by the treating physician. Included patients cannot be randomized before 6 weeks after symptom onset since spontaneous early recovery occurs in some patients [4]. In daily practice, management ranges from early neurolysis to prolonged non-invasive treatment [30]. To reflect daily practice in the study design, the time window for randomization allows for some variation. The Randomization Module within REDCap will be used (web-based randomization/treatment allocation system) to generate an unpredictable allocation sequence, as designed by the trial statistician. Randomization will not be stratified by centre because of the risk of selection bias due to treatment predictability in centres with low inclusion rates.

### Interventions

The patient will be randomized in two arms: (1) surgical management by means of neurolysis of the peroneal nerve and (2) non-invasive treatment.

#### Surgical management

Neurolysis of the peroneal nerve will be performed in the conventional manner with or without loupe magnification or microscope. The procedure can be performed under local, locoregional or general anaesthesia. Pneumatic compression to restrict blood flow in the operation area during surgery can be used. The surgical approach is usually through a curvilinear incision just distal to the fibular head. The subcutaneous tissue is bluntly dissected, and the common peroneal nerve is identified proximal to the peroneus longus muscle. The peroneal nerve is then released from the surrounding fibrous tissue and fascia. The anterior intermuscular septum is usually not cut, but this can be done if deemed necessary, or according to local standard practice. The nerve is decompressed distally where it dives under the peroneus longus muscle. The decompression at this site is essential. It is up to the surgeon to decide if decompression beyond the bifurcation is necessary, based on intraoperative findings. Details of the surgical procedure will be recorded in the surgical report. The surgical procedure is not considered very complex. Therefore, every general neurosurgeon is qualified to perform the procedure in participating patients. A neurolysis can also be performed by an orthopaedic surgeon with experience in peripheral nerve surgery. Preferentially, every participating centre has an experienced peripheral nerve surgeon. In practice, one experienced surgeon will perform all procedures.

Per protocol, patients randomized to surgery need to be operated within 1 week after randomization. Due to logistic reasons, surgery is scheduled for all patients after inclusion and cancelled in case a patient is randomized to conservative treatment. Patients are not informed about the scheduling of surgery to avoid creating bias towards surgery. The patient will be hospitalized for up to 2 nights or can be operated on in an ambulatory day-care surgery setting. Postoperative treatment follows standard of care and can include physiotherapy and medication, a decision that is left to the treating physician.

#### Conservative management

Evidence on the conservative management of foot drop is scarce. Despite the lack of studies on physiotherapy to improve foot drop of peripheral origin, a training programme is recommended for every patient with foot drop [33]. This treatment aims to reduce muscle atrophy, preserve ankle mobility and improve gait in general. A basic standard protocol for physiotherapy is proposed. However, the protocol should be adapted to the clinical presentation and needs of every individual patient. Standard instructions for the physiotherapist will be provided and include:

- Mobilization of ankle and foot, stretching of calf muscles (prevention of contractures)
- Tonification of the dorsiflexion- and eversion muscles of the ankle
- Proprioceptive training
- Gait rehabilitation
- Home exercise schedule

The training programme should be progressive. To evaluate compliance, patients will be asked to complete a training diary. Sixty reimbursed sessions of physiotherapy will be prescribed at a frequency of 1/2 sessions per week, with a possibility of a higher frequency during the first months. Based on expert opinion, the use of electrostimulation is neither encouraged nor prohibited. The routine use of an orthosis during the first 6 weeks is not supported. Most patients receive a prefabricated orthosis at this stage. However, this does not always meet the requirements of the patients at later stages. When the foot drop is irreversible, an ankle foot orthosis can help improve everyday mobility.

Cross-over to surgical treatment is not allowed until the primary endpoint at 9 months is reached. After the primary endpoint is reached, a cross-over from the conservative arm to surgery is allowed. This decision will be left at the discretion of the treating physician in discussion with the patient.

### Outcome assessment

Outcome assessors will be blinded to treatment allocation. To facilitate blinding, all patients wear long trousers and apply a bandage at the level of the fibular head, to cover a potential scar. Patients are asked to not discuss their treatment modality with the outcome assessors and are reminded of the blinded measures prior to each study visit. Unblinding is never required, since the patient and treating physician are not blinded. The treating physician can deal with any emergency situation without requiring involvement of the blinded outcome assessor. All outcome assessors will be trained using training videos that are available on the website (www.footdroptrial.com). This training is mandatory in all centres, will reduce interobserver variability and will improve data quality.

Baseline assessments will be collected at the randomization visit. Follow-up examinations by the blinded outcome assessors will take place 6 weeks, 3 months, 6 months, 9 months and 18 months after randomization (Table 2). Patients randomized to surgery will be evaluated 10 days after surgery by the surgeon as well. The primary endpoint is reached 9 months after randomization. The physician will evaluate the patients during the screening and randomization visit, 10 days and 6 weeks after randomization. This is considered standard of care. Study-specific visits will be completed by the blinded outcome assessors.

**Table 2:** Data collection and outcome measures. *d* days, *w* weeks, *m* months

Time since randomization	0	10d	6w	3m	6m	9m	18m
6-min walk test	X		X	X	X	X	X
10-m walk test	X		X	X	X	X	X
Isometric dynamometry	X	X	X	X	X	X	X
Muscle strength (MRC score)	X	X	X	X	X	X	X
Electrodiagnostics				X		X	
Functional ambulation categories	X		X	X	X	X	X
Stanmore questionnaire	X		X	X	X	X	X
Sensory changes	X	X	X	X	X	X	X
EQ5D-5L questionnaire	X	X	X	X	X	X	X
Return to work			X				
Surgical complications		X	X				X
Treatment record	X		X	X	X	X	X
Ability to walk barefoot	X		X	X	X	X	X
Need for orthosis	X		X	X	X	X	X
WPAI questionnaire	X		X		X		

#### Primary endpoint

The primary endpoint is the difference in distance covered in meters during the 6-min walk test (6MWT) between randomization and 9 months after randomization.

Based on 41 patient interviews, gait improvement is crucial in foot drop recovery. 88% of interviewed patients related treatment success to gait improvement. The 6MWT has been used in other studies examining gait in patients with foot drop due to other pathologies including multiple sclerosis (MS) and stroke [34–40]. Since the 6MWT tests speed and distance over a longer period of time, ankle dorsiflexion fatigue is taken into account as well. The 6MWT will be performed twice at each study visit, to take the learning effect into account.

The minimal age- and sex-specific normal 6-minute walking distance (6MWD) is defined as 82% of the applied reference equations for prediction of the 6MWD [41, 42]. The reference value that will be used is 6MWD_pred_(m) = 868.8 − (age_years_ × 2.99) − (gender × 74.7). The value for gender is 0 in male subjects and 1 in female subjects [42]. Based on the available data in the stroke and MS literature, the reference values in a normal population and other reports [43], we estimate the minimal clinically important difference in 6MWD in patients with foot drop due to peroneal nerve entrapment to be 10% of the minimal predicted age- and sex-specific reference value for that patient.

#### Key secondary endpoint

##### Time to recovery

Limited data are available regarding time to recovery after surgical or conservative treatment in peroneal nerve entrapment [4]. Time to recovery is defined as the time necessary to cover the minimal age- and sex-specific normal 6MWD and the time necessary for foot drop recovery to an MRC score ≥ 4 for ankle dorsiflexion.

#### Secondary endpoints

Ankle dorsiflexion strength Gait impairment at 6 weeks, 3 months, 6 months, 9 months and 18 months after randomization.

Ankle dorsiflexion strength will be measured by the MRC score. The MRC score is a clinical assessment and has consistently been applied for the assessment of patients with peroneal nerve entrapment in the literature [2, 13, 21]. A score from 0 to 5 is used to grade the strength of a muscle group in relation to the maximum expected for that muscle (see Table 3). The MRC score is a widely used tool to assess muscle strength in peripheral nerve pathology and is used on a daily basis around the world to assess the strength of foot dorsiflexion in patients with foot drop. Training videos for the trial investigators are made available, so that the MRC scoring will be more rigorously applied and inter-observer variability will be reduced to a strict minimum. The additional use of dynamometry allows to document ankle dorsiflexion strength in an objective manner. Dynamometry has frequently been used to assess muscle strength in patients with foot drop [44–46].

**Table 3:** The Medical Research Council (MRC) muscle strength scale

Medical research council (MRC) muscle strength	
MRC 0	No muscle contraction
MRC 1	Muscle contraction, no movement
MRC 2	Movement, but not against gravity
MRC 3	Movement against gravity, but not against resistance
MRC 4	Movement against resistance, but not normal strength
MRC 5	Normal strength

##### Gait impairment at 6 weeks, 3 months, 6 months, 9 months and 18 months after randomization

A thorough gait assessment including 2 questionnaires (functional ambulation categories (FAC), Stanmore questionnaire), gait speed during the 10-m walk test and the recording of the ability to walk barefoot and the need for ankle orthosis.

FAC is a six-point scale (FAC 0 = non-functional to FAC 6 = independent on (non-) level surfaces), that can help determine how much assistance a patient requires. A FAC ≥ 3 has been considered a surrogate of general walk ability in assessing mobility in stroke patients [47, 48]. In the FOOTDROP trial, a good outcome correlates with a FAC of 5. The Stanmore questionnaire consists of seven sections (pain, need for orthosis, normal shoes, functional outcome, muscle power, degree of active dorsiflexion and foot posture) adding up to a sum score of 100 points [49]. The outcome can be classified as excellent (85–100), good (70–84), fair (55–69) and poor (< 55).

Furthermore, the proportion of patients in both groups who reach minimal normal age- and sex-specific reference values for 6MWD 9 months after randomization, as well as the difference in distance covered in meters during the 6-min walk test between baseline and 6 weeks, 3 months, 6 months and 18 months after randomization will be recorded.

##### Surgical complications at 10 days, 6 weeks and 18 months

Overall, neurolysis of the peroneal nerve is considered a low-risk surgery [50]. A list of possible complications based on the available literature [1, 2, 13, 21, 24, 51–53] and investigator experience is made (Table 4) to score complications uniformly among participating centres.

**Table 4:** Predefined possible surgical complications

Impaired/prolonged wound healing	
Wound infection with need for antibiotics	
Wound dehiscence without need for revision surgery	
Wound dehiscence with need for revision surgery	
Postoperative hematoma without need for revision surgery	
Postoperative hematoma with need for revision surgery	
Transection (partial) of peroneal nerve with new sensory deficit	
Transection (partial) of peroneal nerve with new motor deficit	
Persisting pain in the operated area	
Development of complex regional pain syndrome	

##### Motor and sensory changes at 10 days, 6 weeks, 3 months, 6 months, 9 months and 18 months after randomization

Motor changes in muscles innervated by the peroneal nerve will be evaluated throughout the trial. Ankle eversion and hallux extension strength will be assessed using the MRC score. Sensory changes (light touch, sharp-dull sensation) in the skin innervated by the peroneal nerve will be assessed and, if present, recorded as complete recovery, partial recovery or no recovery.

##### Health-related quality of life (EQ5D-5L) at 10 days, 6 weeks, 3 months, 6 months, 9 months and 18 months after randomization

The EQ-5D 5L is a generic measure of patient-reported health-related quality of life, consisting of two parts [54]. The first part uses five different dimensions to score the quality of life: mobility, self-care, activities of the daily life, pain/discomfort and anxiety/depression. There are five different answer possibilities within each dimension, i.e., “no problem”, “slight problem”, “moderate problem”, “severe problem” and “unable to”. The second part uses a visual analogue scale (VAS) to score the current health status of the patient, ranging from zero to one hundred.

##### Health-economic evaluation of foot drop due to peroneal nerve entrapment

Currently, data about cost-effectiveness are scarce. The FOOT DROP trial will allow for a first health economic analysis of foot drop due to peroneal nerve entrapment. Cost-effectiveness will be calculated as cost per quality-adjusted life-year (QALY) gained using the quality of life data generated by the EQ5D-5L questionnaire throughout the study. Furthermore, return to work at 6 weeks after randomization will be assessed. Finally, patients will complete the Work Productivity and Activity Impairment (WPAI) questionnaire [55] at baseline and at 6 weeks and 6 months after randomization. The WPAI allows for the assessment of employment and professional productivity among other features.

##### Electrodiagnostics (EDX) at 3 months and 9 months after randomization

Electrodiagnostic studies will include nerve conduction studies and electromyography (EMG) [56]. The following EDX parameters will be recorded: difference in conduction velocity across the fibular head, the presence of a conduction block at the level of the fibular head, rest and voluntary EMG abnormalities and signs of axonal or mixed damage.

### Feasibility pilot study

To assess the feasibility of the study design as described in this manuscript, the authors conducted a feasibility pilot study in 5 centres in Belgium and 1 centre in the Netherlands. The main objective of the feasibility study was to evaluate if enough eligible patients were willing to participate in the trial. No analysis of the trial endpoints took place. Pilot study data will be integrated in the full study data set. The pilot study ran between 29 April 2021 and 16 October 2022 and was successfully concluded. A feasibility pilot study overview will be submitted for publication in the near future.

### Protocol adherence

Patients will receive a message prior to each evaluation moment, to remind them of the upcoming assessments. At every assessment, the schedule of the assessments to come will be discussed with the patients as an extra reminder. If visits or data collection time-points are missed, despite previously mentioned efforts, the following measures will be taken:

- The outcome assessor will inform the treating physician, PI, clinical trial assistant or any other non-blinded study personnel. The patient will be contacted to reschedule the visit as soon as possible. This cannot be done by the outcome assessor to guarantee blinding.
  - ○ If the patient is not able to reschedule, the reason will be documented in the electronic case report file (eCRF). If possible, certain assessments will be evaluated through telephone: EQ-5D 5L questionnaire, WPAI questionnaire, sensory changes, ability to walk barefoot, need for foot-ankle orthosis, return to work and registration of treatment
- The scheduling of the following appointments will be checked with the subject, to be sure that further follow-up is guaranteed.

The site and study team must take preventive measures to avoid a subject being lost to follow-up (at least 3 contacts (e.g., telephone calls or e-mails) must be placed to the last available telephone number or e-mail address) and 1 registered letter must be sent by post to the last available home address. If the subject is still unreachable after all contact attempts listed above, he/she will be considered to be lost to follow-up.

If a patient decides to not attend to some follow-up visits, efforts should be made to convince the patient to agree to the study visit at 9 months after randomization. The visit at 9 months is the most important study visit due to assessment of the primary endpoint. In this way, the patient is not lost to follow-up and the other visits will be marked as ‘missed data collection time-points’. For protocol non-adheres, all endpoints will be assessed according to protocol if the patient chooses to remain included in the trial.

### Sample size

Given the fact that no reliable estimates of the 6MWD are available in this population, the sample size calculation is performed assuming a moderate effect size (mean difference divided by the standard deviation). Based on an effect size of 0.5, 172 patients would have 90% power to show a significant difference at a two-sided significance level of 5% in the change in the 6MWD from baseline to 9 months after randomization using a t-test. The sample size calculation was performed with G*Power, version 3.1.9.4. To compensate for an expected 5% dropout, a total of 182 patients (91 patients per group) will be included. The expected dropout rate of 5% is based on the results of patient interviews and a large, multi-centre, randomized controlled trial comparing surgery versus non-surgery (or delayed surgery) for patients with radiculopathy caused by lumbar disc herniation with a design very similar to the foot drop trial [57].

A blinded sample size reassessment by the trial statistician is planned when 50% of patients have reached their 9-month visit or 1 month before the final patient would be randomized, whichever occurs first. It will be based on the estimation of the overall variance of the primary outcome for the patients for which the primary endpoint data are available and the minimal clinical difference determined from all patients randomized at that point. The maximum number of patients to be potentially added will be determined before the analysis will take place. Given that a blind analysis will be performed, the type I error will be preserved and no adjustment to the alpha level for the final analysis will be necessary.

### Statistical analysis

The primary analysis set will be the full analysis set (FAS) which will include all patients randomized. Patients will be analysed in the group to which they were randomized, irrespectively which treatment was received, if any. In addition, a per-protocol analysis set will be used for sensitivity analyses.

The primary endpoint will be analysed using a constrained longitudinal data analysis taking the correlation between these measurements into account. The value at baseline and all follow-up measurements will be taken as responses. Indicators for baseline, follow-up visits, treatment group and the interaction between follow-up visits and treatment group will be included in the model. Missing data are appropriately taken into account with this model under the missing at random assumption. This is applicable to no-shows (patients that do not show up at study visits). For patients refusing to participate in the 6MWT, zero as value will be recorded. The primary analysis is the comparison between the two treatment groups of the difference in 6MWD between 9 months and baseline. The mean difference between the two treatment groups with a 95% confidence interval and a two-sided *p*-value will be reported. Superiority of surgery above conservative treatment will be claimed if *p* < 0.05 and the patients in the surgery arm improved more than the patients in the conservative arm.

Several sensitivity analyses will be performed. The first sensitivity analysis is similar to the primary analysis but with follow-up measurements after surgery not being used in the analysis for patients randomized to conservative treatment but who underwent surgery within 9 months. Similarly, follow-up measurements of patients randomized to surgery who did not undergo surgery will not be used in the analysis. This will allow to estimate the treatment effect most honestly. In the unlikely event that in the conservative treatment arm more than 10% cross-overs are observed, this sensitivity analysis will become the primary analysis and the primary analysis the first sensitivity analysis.

A second sensitivity analysis will be a multiple imputation analysis in which missing follow measurements will be imputed according to the conservative treatment arm for both the surgical and conservative treatment arm.

The study is only powered for the primary endpoint but, if the primary endpoint should show a significant difference, it is anticipated that also the timing of recovery, the key secondary endpoint, might yield a significant effect. It will be analysed using a generalized log-rank test with constant weights over time for interval-censored data. In addition, the Turnbull estimator with 95% confidence intervals will be produced. The proportion of patients which recovered will be estimated at each follow-up visit. We do not anticipate any mortality due to the pathology. In case a patient should die, the patient will be censored at his day of death. Full details for the analysis of the primary and all secondary endpoints will be made available in a statistical analysis plan, which will be finalized before database lock.

### Recruitment strategy

A strong, multidisciplinary collaboration in all centres is essential for trial success. To be able to identify all eligible patients, physicians of several disciplines (neurosurgery, neurology, physical medicine and rehabilitation, orthopaedic surgery, reconstructive and plastic surgery) need to be on high alert. Therefore, a motivated local study team reminding and stimulating all physicians involved is essential. To identify suitable trial centres, a feasibility questionnaire was designed with experts from an independent global consultancy agency (Keyrus). The estimated recruitment rate will be based on the feasibility questionnaire and results from the internal feasibility pilot study. Per year, we defined the number of patients that we minimally expect to randomize. If this specific threshold is not reached, we can escalate recruitment by initiating the study in additional centres.

### Trial supervision and responsibilities

The role of the Trial Steering Committee (TSC) is to provide the overall supervision of the trial. The TSC monitors trial progress and conducts and advises on scientific credibility. The TSC will consider and act, as appropriate, and ultimately carries the responsibility for deciding whether a trial needs to be stopped on grounds of safety or efficacy. The TSC will meet on average 3 times per year the first year and twice a year thereafter. The TSC is composed of the Chief Investigator (CI), the trial statistician, the trial Project Manager (PM), an independent expert, a representative of other participating centres or groups, up to 2 patients or members of the public, 1 representative of the sponsor, and 1 representative of the funder.

The day-to-day management of the study will be performed by the Trial Management Group (TMG), which is distinct from the TSC. The TMG is composed of the CI, the PM, the data manager, the co-chief investigator for the French-speaking centres, the trial statistician and the sponsors’ clinical trial assistant (CTA). The TMG will be responsible for data analysis and interpretation as well as writing the report. Submission for publication will occur in consultation with the principal investigators and study funder (KCE).

The Trial Data Manager will perform extensive consistency checks on the received data. Queries will be issued in case of inconsistencies in accordance with internal procedures. A Data Management Plan is developed to map data flows, data validation measures that will be taken and how (interim) database lock(s) will be managed. Data in the eCRF will be pseudonymized. Only a site-specific and confidential Subject Identification Log provides the link between named subject source records and the pseudonymized data set. All source data will be kept at a secured location with restricted access at all times. These data must be collected and processed with adequate precautions to ensure confidentiality and compliance with applicable data protection laws and regulations and more in particular the EU General Data Protection Regulation 2016/679 (GDPR) and relevant national laws implementing the GDPR.

Based on the European Medicines Agency (EMA) guidelines on data monitoring committees, the organization of a Data Safety Monitoring Board is not required for the FOOTDROP trial. The independent experts in the TSC will act as an ‘extern advisory board’ to follow up on possible harm (e.g. related to late cross-over to surgery) and to follow up on blinding and accidental unblinding. The risks associated with trial participation are comparable to risks of standard medical care, since both treatment arms are considered standard of care. The participant will be asked to report any adverse event related to the study-specific intervention to the study team. These reported events will be documented by the investigator in the source documents.

### Trial monitoring

Trial monitoring will be conducted by trained study monitors from the Clinical Trial Center of University Hospitals Leuven. There are no competing interests to report. Prior to the start of the study, all required approvals must be obtained. During the study a monitor can contact and visit the site and must be permitted, on request, to access the study facilities and all source documents needed to verify adherence to the protocol and the completeness, consistency and accuracy of the data being entered in the CRF. The processes reviewed can relate to participant enrolment, consent, eligibility, and allocation to trial groups; adherence to trial interventions and policies to protect participants, including reporting of harm and completeness, accuracy, and timelines of data collection. Monitoring can be done by exploring the trial dataset or by performing site visits. A detailed trial monitoring plan is available at request. The Investigator (and head of institution, if required by regional regulations) will make source data and documents for this study available to an appropriately qualified quality assurance auditor mandated by the sponsor or to regulatory authority inspectors.

### Access to data and dissemination policy

The study results will be owned by the party who generates them. The sponsor will have access to the study data. Access to study data by KCE is fully defined in the contract between KCE and the sponsor and the research agreement template is publicly available on the KCE website (https://kce.fgov.be/en/kce-trials/calls). After the completion of the study, the sponsor will transfer the pseudonymized study data set to KCE, if required for health economic analysis. The study data shall not be provided to a third party without the prior written approval of KCE.

Publications will be coordinated by the CI. Authorship to publications will be determined in accordance with the requirements published by the International Committee of Medical Journal Editors and in accordance with the requirements of the respective medical journal. It is anticipated that the primary results of the overall Trial shall be published in a multi-centre publication. There is no intended use of professional writers. Participating Sites are not allowed to publish any subset data or results from the Trial prior to such multi-centre publication. Any publication by a Participating Site must be submitted to the sponsor for review at least thirty [30] calendar days prior to submission or disclosure. Sponsor shall have the right to delay the projected publication for a period of up to 3 months from the date of first submission to the sponsor in order to enable the sponsor to take steps to protect its intellectual property rights and know-how.

The results of the study will be communicated with the participants after all participants have reached the primary endpoint. This data will not be shared with participants until the data interpretation has undergone peer review. Research results should be offered in a timely manner and will therefore be communicated within 1 month after the data are peer reviewed.

## Discussion

This manuscript discusses the design and rationale of a randomized controlled trial on the treatment of foot drop due to peroneal nerve entrapment. The main objective is to assess whether foot drop, caused by peroneal nerve entrapment recovers better within 9 months after decompressive surgery compared to prolonged conservative treatment. This research question is highly relevant, since peroneal nerve entrapment is one of the most frequent causes of foot drop, causing gait difficulties and increased risk of falling [3, 58]. In the absence of guidelines or high-quality literature, current management strategies range from early neurolysis to prolonged non-invasive care without surgery. No standard of care is established thus far [4, 30]. The FOOTDROP trial will be the first randomized controlled trial to compare non-invasive and surgical treatment for foot drop due to peroneal nerve entrapment in a methodologically sound manner [4]. The trial is designed to mirror daily practice. To accommodate for some variation in timing of surgical treatment, the time window for randomization covers a period of 8 weeks. Furthermore, the protocol allows for some variability in intensity and frequency of physiotherapy. Since spontaneous recovery after onset of foot drop does occur [4], randomization to surgery is only possible after 10 (± 4 weeks) after onset of foot drop. Cross-over from non-invasive treatment to surgical decompression of the peroneal nerve is not allowed before 9 months after randomization which is the timing of the primary endpoint, to account for possible differences in time to recovery between the two treatment strategies. The intended sample size of the study population is believed to be sufficiently large to detect clinically meaningful differences between both treatment strategies. Trial results will hopefully aid physicians in future decision-making about the preferable treatment of patients with foot drop due to peroneal nerve entrapment.

### Trial status

A internal, feasibility pilot study ran in 6 centres in Belgium and the Netherlands between 29 April 2021 and 16 October 2022. Ethics committee approval was given in all centres involved. The feasibility pilot study was successfully completed and shaped the protocol as discussed in this manuscript (version 2.0, version date 30-10-2022). Overall, 19 patients were included, of which 15 patients were randomized. The protocol essentially does not differ from the internal feasibility pilot study. Two trial assessments were abandoned after the pilot study: the 36-Item Short Form Health Survey (SF-36) and the assessment of ankle dorsiflexion range of motion. The SF-36 was found to be too extensive and not providing any added value on top of the EQ5D-5L questionnaire. Assessment of the range of motion was very dubious and difficult to organize in a uniform way and failed during the pilot study. A manuscript discussion of the pilot study will be submitted for publication in the near future.

The full study will run for approximately 5.5 years. We expect to include the first patient of the full study in April 2023 and the last patient 4 years later (April 2027). Data from the pilot study will be incorporated into the full study database.

## Supplementary Information

**Additional file 1: Appendix 1.** SPIRIT Checklist.

**Additional file 2: Appendix 2.** The World Health Organization Trial Registration Data Set for the FOOTDROP study.

**Additional file 3: Appendix 3.** Informed consent form FOOTDROP trial.

## Data Availability

The study results will be owned by the party that generates them. The sponsor (University Hospitals Leuven) will have access to the study data. At the end of the study, KCE will receive from sponsor-specific study data. This will only be anonymous study data or, where requested by KCE, coded personal data are made available to KCE. The study data shall not be provided to a third party without the prior written approval of KCE, which approval KCE shall not unreasonably withhold or delay and which KCE may subject to specific conditions in order to ensure that the provision of said study data does not have a negative impact on the further performance of the study, the rights granted to KCE under the research agreement and/or the benefit of the Study for the patients and/or the public payers.

## Abbreviations

FOOTDROP: Follow-up and Outcome of Operative Treatment with Decompressive Release Of The Peroneal nerve
KCE: Belgian Health Care Knowledge Centre
MRC: Medical Research Council
EQ5D-5L: 5 Level EUROQoL – 5 Dimensions
SF-36: 36-Item Short Form Health Survey
6MWT: Six-minute walk test
6MWD: Six-minute walking distance
10MWT: Ten-meter walk test
WPAI: Work Productivity and Activity Impairment Questionnaire
KCE: Belgian Health Care Knowledge Center
EDX: Electrodiagnostics
EMG: Electromyography
MRI: Magnetic resonance imaging
FAC: Functional ambulation categories
QALY: Quality-adjusted life-year
MS: Multiple sclerosis
VAS: Visual analogue Scale
TSC: Trial Steering Committee
CI: Chief investigator
PM: Project manager
TMG: Trial Management Group
CTA: Clinical trial assistant
