# Supplementary material for: Conservative versus surgical treatment of foot drop in peroneal nerve entrapment: rationale and design of a prospective, multi-centre, randomized parallel-group controlled trial
Source: Trials. 2022 Dec 30;23:1065. doi: 10.1186/s13063-022-07009-x (PMC9801603; doi:10.1186/s13063-022-07009-x)
Supplement: Supplementary file 1 — Additional file 1: Appendix 1. SPIRIT Checklist. [file 13063_2022_7009_MOESM1_ESM.doc]

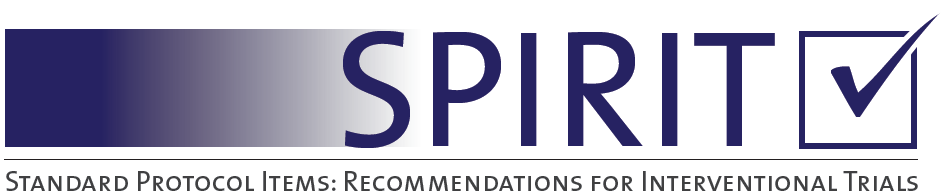


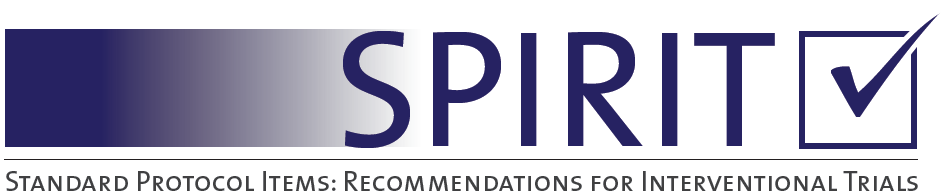
**Appendix 1: The SPIRIT 2013 Checklist for the FOOTDROP study.**

**Colour legend:**

- Criterion fulfilled
- Criterion partially fulfilled
- Criterion not fulfilled
- Criterion not applicable

SPIRIT 2013 Checklist: Recommended items to address in a clinical trial protocol and related documents*

| Section/item | ItemNo | Description |
| --- | --- | --- |
| **Administrative information** | | |
| Title | 1 | Descriptive title identifying the study design, population, interventions, and, if applicable, trial acronym  **Included in the manuscript, page 3 (background).** |
| Trial registration | 2a | Trial identifier and registry name. If not yet registered, name of intended registry  **Included in the manuscript, page 4 (trial registration).** |
|  | 2b | All items from the World Health Organization Trial Registration Data Set  **Relevant items for the readers of this protocol are included throughout the manuscript. The World Health Organization Trial Registration Data Set is included as appendix 2.** |
| Protocol version | 3 | Date and version identifier  **Version and version date are included in the manuscript, page 15 (trial status).** |
| Funding | 4 | Sources and types of financial, material, and other support  **The KCE is acknowledged as study sponsor in the manuscript, page 17 (declaration). Data collection, analysis and interpretation will be the responsibility of the study team and trial statistician. KCE will oversee the process through monthly status meetings. The study team will write the manuscript and will take feedback from the KCE into account.** |
| Roles and responsibilities | 5a | Names, affiliations, and roles of protocol contributors  **Included in the title page of the manuscript. Contributions are explained on page 17, declaration.** |
|  | 5b | Name and contact information for the trial sponsor  **Included in the title page of the manuscript (page1).** |
|  | 5c | Role of study sponsor and funders, if any, in study design; collection, management, analysis, and interpretation of data; writing of the report; and the decision to submit the report for publication, including whether they will have ultimate authority over any of these activities  **Included in the manuscript, page 12 and 13 (trial supervision and responsibilities). This topic is also discussed in the declaration section, page 17).** |
|  | 5d | Composition, roles, and responsibilities of the coordinating centre, steering committee, endpoint adjudication committee, data management team, and other individuals or groups overseeing the trial, if applicable (see Item 21a for data monitoring committee)  **Included in the manuscript page 12 and 13 (trial supervision/responsibilities and trial monitoring).** |
| Introduction |  |  |
| Background and rationale | 6a | Description of research question and justification for undertaking the trial, including summary of relevant studies (published and unpublished) examining benefits and harms for each intervention  **This is discussed in the background section, page 3. This is also extensively discussed in the study protocol (background, page 14-17).** |
|  | 6b | Explanation for choice of comparators  **Both are standard of care, as discussed in the background section, page 3. This is also discussed in the study protocol (rationale, page 18-19 & background, page 14-17).** |
| Objectives | 7 | Specific objectives or hypotheses  **Included in the study protocol, page 4 (methods section).** |
| Trial design | 8 | Description of trial design including type of trial (eg, parallel group, crossover, factorial, single group), allocation ratio, and framework (eg, superiority, equivalence, noninferiority, exploratory)  **Included in the methods section page 4 and statistical analysis section page 11 and 12.** |
| Methods: Participants, interventions, and outcomes | | |
| Study setting | 9 | Description of study settings (eg, community clinic, academic hospital) and list of countries where data will be collected. Reference to where list of study sites can be obtained  **Included in the study protocol page 4 (patients).** |
| Eligibility criteria | 10 | Inclusion and exclusion criteria for participants. If applicable, eligibility criteria for study centres and individuals who will perform the interventions (eg, surgeons, psychotherapists)  **Eligibility criteria are included in the manuscript on page 5 (table 1). Qualifications for the surgeon are eluded on page 6.** |
| Interventions | 11a | Interventions for each group with sufficient detail to allow replication, including how and when they will be administered  **Included in the manuscript, page 5, 6 and7 (surgical and conservative management).** |
|  | 11b | Criteria for discontinuing or modifying allocated interventions for a given trial participant (eg, drug dose change in response to harms, participant request, or improving/worsening disease)  **We included no no-cross over policy from conservative treatment to surgery in the manuscript, page 7.** |
|  | 11c | Strategies to improve adherence to intervention protocols, and any procedures for monitoring adherence (eg, drug tablet return, laboratory tests)  **Manuscript page 6: we introduce the training diary to evaluate compliance to non-invasive treatment.** |
|  | 11d | Relevant concomitant care and interventions that are permitted or prohibited during the trial  **Manuscript page 6: it is stated that the trial should mirror daily practice and therefore are all non-invasive treatment strategies allowed (albeit not encouraged, see manuscript). The no cross-over policy to surgery is discussed.** |
| Outcomes | 12 | Primary, secondary, and other outcomes, including the specific measurement variable (eg, systolic blood pressure), analysis metric (eg, change from baseline, final value, time to event), method of aggregation (eg, median, proportion), and time point for each outcome. Explanation of the clinical relevance of chosen efficacy and harm outcomes is strongly recommended  **Included in the manuscript, page 7-10 (section outcome assessment).** |
| Participant timeline | 13 | Time schedule of enrolment, interventions (including any run-ins and washouts), assessments, and visits for participants. A schematic diagram is highly recommended (see Figure)  **Included in the manuscript, see figure 1 page 4 and table 2 page 7.** |
| Sample size | 14 | Estimated number of participants needed to achieve study objectives and how it was determined, including clinical and statistical assumptions supporting any sample size calculations  **This information is included in the manuscript, page 10-11-12 (sample size and statistical analysis).** |
| Recruitment | 15 | Strategies for achieving adequate participant enrolment to reach target sample size  **Included in the manuscript, page 12 (recruitment strategy).** |
| **Methods: Assignment of interventions (for controlled trials)** | | |
| Allocation: |  |  |
| Sequence generation | 16a | Method of generating the allocation sequence (eg, computer-generated random numbers), and list of any factors for stratification. To reduce predictability of a random sequence, details of any planned restriction (eg, blocking) should be provided in a separate document that is unavailable to those who enrol participants or assign interventions  **Included in the manuscript, page 5 (treatment allocation).** |
| Allocation concealment mechanism | 16b | Mechanism of implementing the allocation sequence (eg, central telephone; sequentially numbered, opaque, sealed envelopes), describing any steps to conceal the sequence until interventions are assigned  **Included in the manuscript, page 5 (treatment allocation).** |
| Implementation | 16c | Who will generate the allocation sequence, who will enrol participants, and who will assign participants to interventions  **Included in the manuscript, page 5 (treatment allocation).** |
| Blinding (masking) | 17a | Who will be blinded after assignment to interventions (eg, trial participants, care providers, outcome assessors, data analysts), and how  **Included in the manuscript, page 7 (outcome assessment)** |
|  | 17b | If blinded, circumstances under which unblinding is permissible, and procedure for revealing a participant’s allocated intervention during the trial  **This checklist item is not entirely applicable since neither physician nor patients are blinded to treatment allocation. There are no realistic emergency circumstances that require unblinding of the outcome assessors. This is explained in the manuscript, page 7 (outcome assessment).** |
| **Methods: Data collection, management, and analysis** | | |
| Data collection methods | 18a | Plans for assessment and collection of outcome, baseline, and other trial data, including any related processes to promote data quality (eg, duplicate measurements, training of assessors) and a description of study instruments (eg, questionnaires, laboratory tests) along with their reliability and validity, if known. Reference to where data collection forms can be found, if not in the protocol  **Assessment and collection of trial data is discussed in the manuscript, page 7 to 10 (outcome assessment). Training of assessors is also discussed in this section.** |
|  | 18b | Plans to promote participant retention and complete follow-up, including list of any outcome data to be collected for participants who discontinue or deviate from intervention protocols  **Included in the manuscript, page 10 (protocol adherence)** |
| Data management | 19 | Plans for data entry, coding, security, and storage, including any related processes to promote data quality (eg, double data entry; range checks for data values). Reference to where details of data management procedures can be found, if not in the protocol  **Included in the manuscript, page 12-13 (trial supervision and responsibilities).** |
| Statistical methods | 20a | Statistical methods for analysing primary and secondary outcomes. Reference to where other details of the statistical analysis plan can be found, if not in the protocol  **Included in the manuscript, page 10, 11 and 12 (statistical analysis). It is stated that a statistical analysis plan will be made.** |
|  | 20b | Methods for any additional analyses (eg, subgroup and adjusted analyses)  **Included in the manuscript, page 10, 11 and 12 (statistical analysis).** |
|  | 20c | Definition of analysis population relating to protocol non-adherence (eg, as randomised analysis), and any statistical methods to handle missing data (eg, multiple imputation)  **Included in the manuscript, page 10, 11 and 12 (statistical analysis).** |
| **Methods: Monitoring** | | |
| Data monitoring | 21a | Composition of data monitoring committee (DMC); summary of its role and reporting structure; statement of whether it is independent from the sponsor and competing interests; and reference to where further details about its charter can be found, if not in the protocol. Alternatively, an explanation of why a DMC is not needed  **Included in the manuscript, page 13 (trial monitoring).** |
|  | 21b | Description of any interim analyses and stopping guidelines, including who will have access to these interim results and make the final decision to terminate the trial  **A blinded sample size reassessment is described on page 11 of the manuscript. No other interim analyses are planned. No stopping guidelines were defined in the protocol.** |
| Harms | 22 | Plans for collecting, assessing, reporting, and managing solicited and spontaneously reported adverse events and other unintended effects of trial interventions or trial conduct  **This is discussed on page 12-13 of the manuscript (section trial supervision and responsibilities).** |
| Auditing | 23 | Frequency and procedures for auditing trial conduct, if any, and whether the process will be independent from investigators and the sponsor  **Briefly discussed in the manuscript, page 13 (trial monitoring)** |
| Ethics and dissemination | | |
| Research ethics approval | 24 | Plans for seeking research ethics committee/institutional review board (REC/IRB) approval  **This is stated in the declaration section, page 17.** |
| Protocol amendments | 25 | Plans for communicating important protocol modifications (eg, changes to eligibility criteria, outcomes, analyses) to relevant parties (eg, investigators, REC/IRBs, trial participants, trial registries, journals, regulators)  **There are only plans to communicate important modifications with the participating centres through monthly newsletters. This is briefly discussed in the declaration section, page 17. No other plans have been established.** |
| Consent or assent | 26a | Who will obtain informed consent or assent from potential trial participants or authorised surrogates, and how (see Item 32)  **This is discussed on page 4 and 5 (patients’ section).** |
|  | 26b | Additional consent provisions for collection and use of participant data and biological specimens in ancillary studies, if applicable  **This is not applicable to the FOOTDROP study since there are neither ancillary studies nor biological specimens.** |
| Confidentiality | 27 | How personal information about potential and enrolled participants will be collected, shared, and maintained in order to protect confidentiality before, during, and after the trial  **The use of pseudonymized data and a subject identification log is discussed in trial supervision and responsibilities (page 12-13). This is extensively discussed in the data handling section (12) of the study protocol itself.** |
| Declaration of interests | 28 | Financial and other competing interests for principal investigators for the overall trial and each study site  **Included in the declaration section of the manuscript, page 17.** |
| Access to data | 29 | Statement of who will have access to the final trial dataset, and disclosure of contractual agreements that limit such access for investigators  **Included in the manuscript, page 13 (access to data and dissemination policy).** |
| Ancillary and post-trial care | 30 | Provisions, if any, for ancillary and post-trial care, and for compensation to those who suffer harm from trial participation  **Not applicable to the FOOTDROP trial. There is no ancillary and post-trial care.** |
| Dissemination policy | 31a | Plans for investigators and sponsor to communicate trial results to participants, healthcare professionals, the public, and other relevant groups (eg, via publication, reporting in results databases, or other data sharing arrangements), including any publication restrictions  **Included in the manuscript, page 13 (access to data and dissemination policy).** |
|  | 31b | Authorship eligibility guidelines and any intended use of professional writers  **Included in the manuscript, page 13 (access to data and dissemination policy).** |
|  | 31c | Plans, if any, for granting public access to the full protocol, participant-level dataset, and statistical code  **There are no plans for granting public access to the full protocol, participant-level dataset and statistical code.** |
| Appendices |  |  |
| Informed consent materials | 32 | Model consent form and other related documentation given to participants and authorised surrogates  **A model consent form in English is available in Appendix 3. A reference to the supplementary materials is added to the manuscript (page 17).** |
| Biological specimens | 33 | Plans for collection, laboratory evaluation, and storage of biological specimens for genetic or molecular analysis in the current trial and for future use in ancillary studies, if applicable  **Not applicable, no biological specimens were collected as part of this trial.** |

*It is strongly recommended that this checklist be read in conjunction with the SPIRIT 2013 Explanation & Elaboration for important clarification on the items. Amendments to the protocol should be tracked and dated. The SPIRIT checklist is copyrighted by the SPIRIT Group under the Creative Commons “[Attribution-NonCommercial-NoDerivs 3.0 Unported](http://www.creativecommons.org/licenses/by-nc-nd/3.0/)” license.
