# Supplementary material for: Conservative versus surgical treatment of foot drop in peroneal nerve entrapment: rationale and design of a prospective, multi-centre, randomized parallel-group controlled trial
Source: Trials. 2022 Dec 30;23:1065. doi: 10.1186/s13063-022-07009-x (PMC9801603; doi:10.1186/s13063-022-07009-x)
Supplement: Supplementary file 2 — Additional file 2: Appendix 2. The World Health Organization Trial Registration Data Set for the FOOTDROP study. [file 13063_2022_7009_MOESM2_ESM.pdf]

Appendix 2: The World Health Organization Trial Registration Data Set for the FOOTDROP study.

**Data category**	**Information**
Primary registry and trial identifying number	ClincialTrials.gov<br>NCT04695834
Date of registration in primary registry	4 January, 2021
Secondary identifying numbers	KCE trials number: KCE19-1232<br>Sponsor study number: S62895
Source(s) of monetary or material support	Belgian Health Care Knowledge Center (KCE)
Primary sponsor	University Hospitals Leuven
Secondary sponsor(s)	N/A
Contact for public queries	Dr. Christophe Oosterbos<br>(christophe.oosterbos@uzleuven.be)
Contact for scientific queries	Dr. Christophe Oosterbos<br>(christophe.oosterbos@uzleuven.be)
Public title	A prospective, multi-center, randomized, parallel-group controlled trial to compare conservative versus surgical treatment of foot drop in peroneal nerve entrapment.
Scientific title	A prospective, multi-center, randomized, parallel-group controlled trial to compare conservative versus surgical treatment of foot drop in peroneal nerve entrapment.
Countries of recruitment	Belgium and the Netherlands
Health condition(s) or problem(s) studied	Foot drop due to peroneal nerve entrapment
Intervention(s)	Intervention: Decompressive release of the peroneal nerve at the level of the fibular head.<br>Control: Maximal (prolonged) conservative treatment with physiotherapy aiming at muscle strengthening and gait rehabilitation.<br>Allowed: use of foot-ankle orthosis / electrostimulation and other therapies reflecting daily practice.
Key inclusion and exclusion criteria	Inclusion criteria:<br>- Written informed consent<br>- EDX-documented peroneal nerve entrapment with persisting (10 ± 4 weeks) foot drop (MRC-score ≤ 3)<br>- Imaging (ultrasound/MRI) performed to exclude a compressive mass<br>- Age ≥ 18 years<br>Exclusion criteria:<br>- Posttraumatic or iatrogenic peroneal nerve injury<br>- Peroneal neuropathy due to a compressive mass (e.g. cyst, tumour)<br>- Peroneal nerve entrapment at other sites than the fibular head<br>- Bilateral peroneal nerve entrapment

	- Patients with mental or physical problems that incapacitate them to participate in a physiotherapy program<br>- Psychiatric illness<br>- Pregnancy<br>- Planned (e)migration within 1 year after randomization to another country<br>- Subjects with previous foot drop<br>- Permanently bedridden subjects<br>- Subjects with neurological or musculoskeletal history which could impact foot drop assessment and/or gait analysis
Study type	This is a prospective, multi-center, randomized, parallel-design study.<br>This is a superiority trial: the goal is to prove superiority of surgery to maximal conservative treatment.<br>Subjects will be randomized 1:1 to surgery or to conservative treatment. No cross-over is allowed until the primary endpoint is assessed at 9 months after randomization. After the primary endpoint is assessed, cross-over to surgery is allowed, with extended follow-up at 18 months after randomization (equals 9 months after primary endpoint).
Date of first enrolment	First patient first visit (pilot study): 29-04-2021<br>First patient first visit (full study): 04-2023 (planned)
Target sample size	Overall, the study will enrol 182 subjects in 2 treatment arms, 91 subjects per arm.<br>The statistical analysis plan will contain a blinded sample size reassessment to verify if the planned sample size is sufficient to show the minimal clinical important difference.
Recruitment status	Recruiting
Primary outcome(s)	Difference in distance (in meters) covered during the six-minute walk test between baseline and 9 months after randomization
(Key) secondary outcomes	Key secondary endpoint:<br>- Time to recovery (defined as the time necessary to cover the minimal age- and sex-specific normal 6MWD AND the time necessary for foot drop recovery to an MRC-score ≥ 4 for ankle dorsiflexion<br>Secondary endpoints:<br>- Ankle dorsiflexion strength at 10 days (surgical group), 6 weeks, 3 months, 6 months, 9 months and 18 months after randomization.

	- Gait assessment at 6 weeks, 3 months, 6 months, 9 months and 18 months after randomization.<br>- Complications and neurological deficits at 10 days (surgical group), 6 weeks, 3 months, 6 months, 9 months and 18 months after randomization.<br>- Health-economic assessment at 6 weeks and 6 months after randomization.<br>- Electrodiagnostics (EDX) at 3 months and 9 months after randomization.<br>- - Patient-reported outcome measures at 10 days (surgical group), 6 weeks, 3 months, 6 months, 9 months and 18 months after randomization.
